# Unraveling the gut microbiome’s contribution to pancreatic ductal adenocarcinoma: mechanistic insights and therapeutic perspectives

**DOI:** 10.3389/fimmu.2024.1434771

**Published:** 2024-07-09

**Authors:** Eileen Tabrizi, Fatemeh Pourteymour Fard Tabrizi, Gehad Mahmoud Khaled, Michael P. Sestito, Saeid Jamie, Brian A. Boone

**Affiliations:** ^1^ Department of Microbiology, Immunology and Cell Biology, School of Medicine, West Virginia University, Morgantown, WV, United States; ^2^ Cancer Institute, West Virginia University, Morgantown, WV, United States; ^3^ Department of Community Nutrition, Faculty of Nutrition and Food Sciences, Tabriz University of Medical Sciences, Tabriz, East Azerbaijan, Iran; ^4^ Department of Biotechnology, School of Sciences and Engineering, American University in Cairo, New Cairo, Cairo, Egypt; ^5^ Department of Surgery, West Virginia University School of Medicine, Morgantown, WV, United States; ^6^ Department of Neurobiology, David Geffen School of Medicine, University of California, Los Angeles, Los Angeles, CA, United States

**Keywords:** pancreatic ductal adenocarcinoma (PDAC), microbiome, immune modulation, dysbiosis, antibiotic therapy, bacteriotherapy

## Abstract

The gut microbiome plays a significant role in the pathogenesis of pancreatic ductal adenocarcinoma (PDAC), influencing oncogenesis, immune responses, and treatment outcomes. Studies have identified microbial species like Porphyromonas gingivalis and Fusobacterium nucleatum, that promote PDAC progression through various mechanisms. Additionally, the gut microbiome affects immune cell activation and response to immunotherapy, including immune checkpoint inhibitors and CAR-T therapy. Specific microbes and their metabolites play a significant role in the effectiveness of immune checkpoint inhibitors (ICIs). Alterations in the gut microbiome can either enhance or diminish responses to PD-1/PD-L1 and CTLA-4 blockade therapy. Additionally, bacterial metabolites like trimethylamine N-oxide (TMAO) and lipopolysaccharide (LPS) impact antitumor immunity, offering potential targets to augment immunotherapy responses. Modulating the microbiome through fecal microbiota transplantation, probiotics, prebiotics, dietary changes, and antibiotics shows promise in PDAC treatment, although outcomes are highly variable. Dietary modifications, particularly high-fiber diets and specific fat consumption, influence microbiome composition and impact cancer risk. Combining microbiome-based therapies with existing treatments holds potential for improving PDAC therapy outcomes, but further research is needed to optimize their effectiveness.

## Introduction

Pancreatic ductal adenocarcinoma (PDAC) is a relatively uncommon cancer, but due to its aggressive nature, elusive symptoms, and limited treatment options, it ranks as the third cause of cancer-related deaths in the United States and the seventh globally (1, 2). PDAC is projected to ascend to the second highest cancer-related deaths by the year 2030 (3). Around 90% of PDAC cases are sporadic, with inherited cases making up the remaining 10%. Age-related PDAC incidence rates are generally higher in industrialized regions globally (4). The majority of patients with PDAC exhibit nonspecific symptoms upon presentation, often at an advanced disease stage where curative surgery is no longer feasible. Despite progress in diagnostic methods and treatment approaches for many cancers, the outlook for PDAC is dismal, with a five-year survival rate below 10% (5).

While research has uncovered specific associations between pancreatic cancer and risk factors such as diet, lifestyle, smoking, alcohol intake, obesity, diabetes, and chronic pancreatitis, the exact etiology for most patients remains unclear. Various oncogenes are linked to PDAC, among these, mutations in the Kirsten rat sarcoma viral oncogene homolog (KRAS) are particularly prevalent, accounting for approximately 93.7% of cases. Additionally, alterations in cyclin-dependent kinase inhibitor 2A (CDKN2A), mothers against decapentaplegic homolog 4 (SMAD4), and tumor protein 53 (TP53) are frequently observed, occurring in approximately 50% each (6). However, unlike many other solid tumors, the quest to identify molecular targets for treating PDAC has encountered obstacles. These challenges arise from considerable genetic diversity within individual tumors and across different tumors. Additionally, the pervasive presence of activating mutations in KRAS, a gene that has proven difficult to target therapeutically, further complicates treatment strategies (7, 8). Therefore, understanding the intricate molecular landscape, the complex tumor microenvironment, and the interplay of genetic and environmental factors driving PDAC progression is crucial for devising effective therapeutic interventions (9, 10).

In recent years, increasing evidence has revealed strong correlations between the incidence of pancreatic cancer (PC) and gut-related factors including gut microbiota translocation, oral microbiota imbalance, dysbiosis, and the presence of toxic metabolite products, all of which also influence its prognosis (11). This complex microecosystem plays a vital role in human physiology by influencing metabolism, regulating the mucosal immune system, facilitating digestion, and maintaining intestinal structure (12). Hence, it is unsurprising that the variations identified in the human gut microbiome could reflect individual lifestyle preferences and behaviors, influencing the concentrations of disease biomarkers in the bloodstream. Moreover, dysbiosis, characterized by microbiota imbalance, can lead to conditions affecting multiple aspects of health, including digestion, immunity, cardiovascular function, respiratory health, and even neurological well-being (13).

The gastrointestinal tract and pancreas are connected via the pancreatic duct, which enters the duodenum at the ampulla, facilitating potential interactions between their respective microbiota. This dynamic interplay can lead to dysbiosis and contribute to diseases resulting from microbiota imbalance or pathogen overgrowth. Further, many patients with PDAC in the pancreatic head present with obstructive jaundice, requiring placement of a stent in the bile duct that traverses the ampulla and may promote bacterial translocation from the gut into the pancreas. Although research on the influence of microbial diversity on PDAC is still in its early phases, emerging evidence suggests a plausible connection between the microbiota and PDAC. The gallbladder is often regarded as an unfavorable environment for bacteria, mainly due to the antimicrobial properties of bile acids. These bile acids possess detergent-like effects that can disrupt bacterial membranes, leading to the dissolution of bacterial cells. However, a healthy gallbladder can host a variety of microbial taxa, encompassing phyla such as Actinobacteria, Bacteroidetes, Firmicutes, and Proteobacteria (14, 15). Furthermore, cultivation studies have indicated a correlation between the insertion of biliary stents and a significant rise in bile colonization, predominantly characterized by species from the duodenal microbiota, such as enterococci (16).

In light of these findings, elucidating the complex interplay between the microbiota, environmental factors, and pancreatic health is essential for advancing our understanding of PDAC etiology and devising effective therapeutic strategies. This review aims to explore the current state of knowledge regarding the role of microbiota in PDAC, identify gaps in understanding, and propose future research directions to address these challenges comprehensively.

## Role of the gut microbiome in health and disease

The gastrointestinal tract of mammals harbors a diverse array of microorganisms, collectively referred to as the gut microbiota. This complex microbial community serves as an essential component in safeguarding the overall health and well-being of the host organism. Beyond mere presence, the gut microbiota actively participates in various physiological processes crucial for host survival and function. Furthermore, emerging research suggests that alterations in the composition and function of the gut microbiota may influence the development and progression of cancer, highlighting its significance in cancer biology.

The human gut microbiota comprises over 100 trillion of microbes from over a thousand different species residing in the gut. In comparison to the approximately 23,000 genes found in the human genome, the gut microbiome boasts over 3 million genes, which can yield thousands of metabolites (17, 18). This indigenous microbial population residing in the intestine serves as a “hidden organ”, facilitating nutrient absorption, regulating epithelial growth, and training the innate immune system (19). The gut microbiota serves as a pivotal regulator of digestion throughout the gastrointestinal tract, with commensal bacteria playing a crucial role in various metabolic processes. These microbes are instrumental in the extraction, synthesis, and absorption of numerous essential nutrients and metabolites, including bile acids, lipids, amino acids, vitamins, and short-chain fatty acids (SCFAs). Moreover, the gut microbiota plays a vital immune function by warding off pathogenic bacterial colonization. This defense mechanism involves inhibiting the growth of harmful bacteria, utilizing available nutrients, and producing bacteriocins to suppress their proliferation. Additionally, the gut microbiota contributes to the maintenance of intestinal epithelial integrity, thereby preventing bacterial invasion and preserving the overall health of the gastrointestinal system (20).

Microorganisms employ various competitive mechanisms to prevent pathogenic colonization, including nutrient metabolism, pH modulation, secretion of antimicrobial peptides, and modulation of cell signaling pathways. Furthermore, recent research has shed light on the pivotal role of commensal bacteria and their byproducts in orchestrating the development, maintenance, and function of both innate and adaptive immune cells. These findings underscore the interplay between the microbiota and the immune system, highlighting the significance of microbial-host interactions in maintaining health and combating diseases (18, 21).

**Figure 1:**
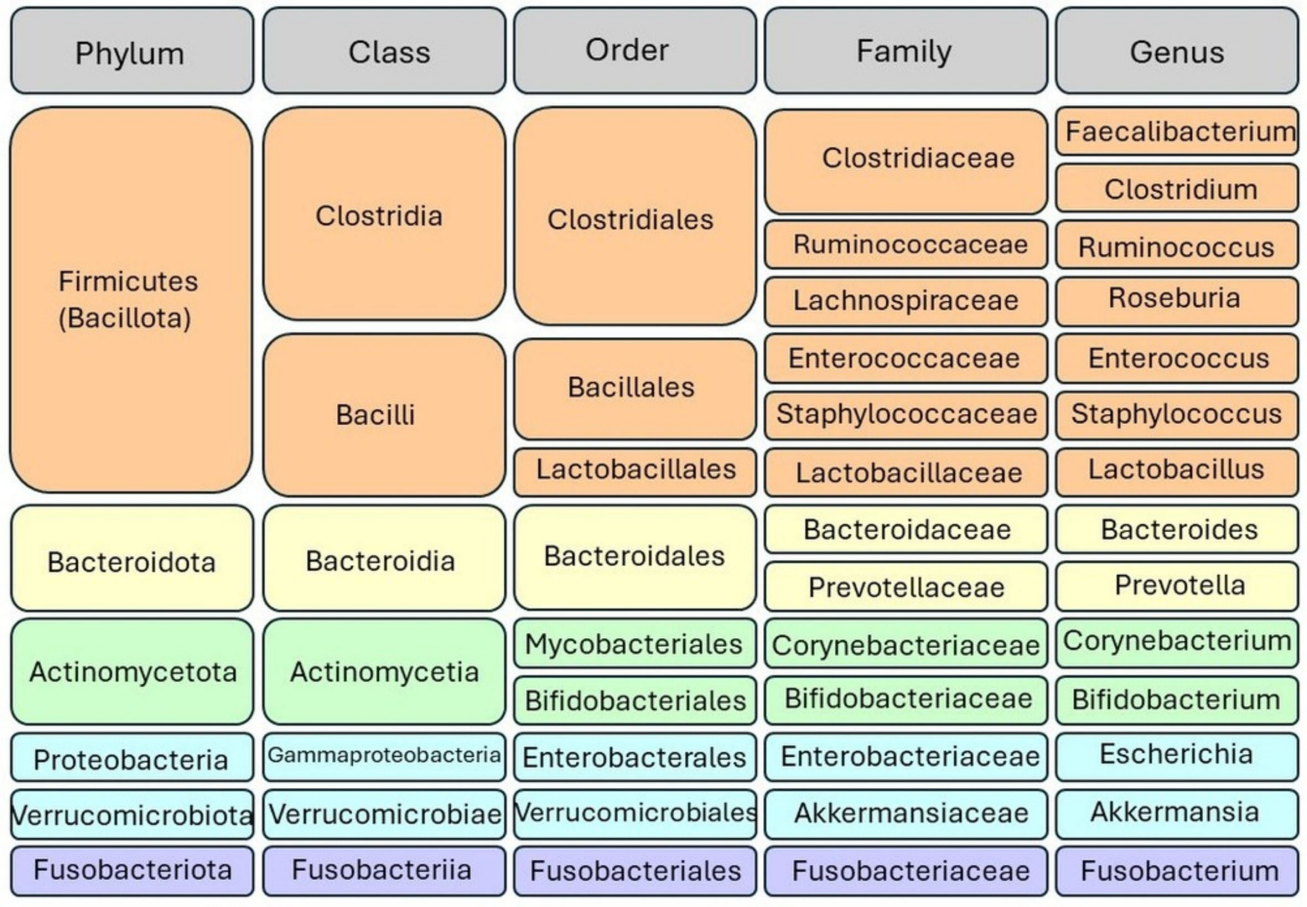
Taxonomic classification of gut microbiota. Bacteria are categorized into hierarchical taxonomic levels including phyla, classes, orders, families, genera, and species. The Firmicutes and Bacteroidetes phyla represent the predominant bacterial taxa in the gut microbiome.

The gut microbiota encompasses a variety of microorganisms, comprising not only bacteria but also yeast and viruses. Taxonomically, bacteria are categorized into hierarchical levels, including phyla, classes, orders, families, genera, and species. The gut microbiota is primarily composed of several dominant phyla, including Firmicutes, Bacteroidetes, Actinobacteria, Proteobacteria, Fusobacteria, and Verrucomicrobia. Among these, Firmicutes and Bacteroidetes stand out as the most prevalent, collectively constituting approximately 90% of the gut microbial population, indicating their significance in shaping the composition and functionality of the gut microbiome; **Figure 1** illustrates the taxonomy of the common gut microbiome. The Firmicutes phylum boasts a diverse array of over 200 distinct genera, including well-known members such as Lactobacillus, Bacillus, Clostridium, Enterococcus, and Ruminicoccus. Among these, the Clostridium genera alone account for a substantial majority, comprising approximately 95% of the Firmicutes phylum. Meanwhile, the Bacteroidetes phylum is characterized by dominant genera such as Bacteroides and Prevotella. In contrast, the Actinobacteria phylum exhibits comparatively lower abundance, with the Bifidobacterium genus serving as its primary representative (18, 22).

The microbiota within mammals exhibits an interplay of substantial diversity and consistency. Upon birth, it undergoes an initial diversification process and subsequently adapts in reaction to changes in diet and health status. Despite these fluctuations, the microbiota also demonstrates a remarkable ability to preserve essential microbial communities over prolonged periods, sometimes persisting for years. This dual nature of variability and stability underscores the resilience and complex dynamics governing the mammalian microbiota (23).

The taxonomic and functional composition of the human gut microbiota undergoes considerable variability across different segments of the gastrointestinal tract. Within the same individual, the gut microbiota can undergo dynamic changes influenced by various factors such as developmental transitions during infancy, aging processes, and environmental influences including antibiotic usage. The diversity of the gut microbiota also varies among individuals due to several factors. One notable factor is the distinction in gut microbiota profile clusters, referred to as enterotypes. There are three primary enterotypes characterized by the prevalence of Prevotella (linked to carbohydrate-rich diets), Bacteroides (associated with high protein and animal fat diets), or Ruminococcus (related to a diet high in resistant starch) (24).

Additionally, factors such as body mass index (BMI) levels play a role in shaping gut microbiota composition, with differences observed between individuals with varying BMI categories. Moreover, external factors such as lifestyle choices, frequency of exercise, ethnicity, and dietary and cultural practices exert considerable influence on the diversity and abundance of gut microbial communities (12, 13, 18). These variations underscore the dynamic nature of the gut microbiome, which adapts and responds to both internal and external cues throughout an individual’s lifespan.

## The gut microbiome in PDAC

While commensal bacteria have been shown to promote a state of homeostasis in several physiological processes, microbial dysbiosis has been implicated in a broad range of human pathologies including inflammatory bowel disease, chronic liver disease, graft versus host disease, and multiple malignancies (25–28). The microbiome has been found to be a contributor to oncogenesis of several intestinal tract malignancies including laryngeal, esophageal, gastric and colorectal cancers (29). Specifically, shifts in oral and gut microbial composition can play a role in PDAC carcinogenesis, immune cell infiltration, response to chemo- and immunotherapy, and overall clinical outcomes. These associations have been made for both overall microbiome signatures and specific microbes. A link between greater microbiome diversity in patients with PDAC and overall survival has been established (30). One study used 16S rRNA gene sequencing of surgically resected PDAC tumors from patient cohorts established at MD Anderson Cancer Center and Johns Hopkins. They found that patients with long-term survival (overall survival (OS) greater than five years) had a greater alpha-diversity, defined as the number of species present within each tumor sample, than stage-matched short-term survival patients (OS less than five years). Further, they identified a microbiome signature (*Pseudoxanthomonas-streptomyces-Saccharopolyspora-Bacillius clausii)* that was highly predictive of survival in multivariate analysis. Similar findings were published on samples obtained from patients in China showing higher intratumor microbiome diversity in long-term survivors (31). Investigators confirmed that dominant phyla in samples included *Proteobacteria, Firmicutes*, and *Bacteroidetes*, similar to the US study. However, they also noted enrichment of *Sphingomonas* and *Megasphaera* in the long-term survivor group that were not seen in the US cohorts. Comparing the two study populations, authors suggested that differences were likely due to a combination genetic and extrinsic factor such as dietary differences. These microbiome signatures may represent an important prognostic biomarker in PDAC patients in the future.

Using murine models to manipulate gut microbiome composition has also provided key insights into its role in PDAC progression. Pushalkar et al. found that both germ-free KC mice and antibiotic-treated orthotopic models exhibited reduced overall tumor burden relative to controls with intact microbiome (32). The transgenic mouse model further demonstrated reduced pancreatic dysplasia and intra-tumoral fibrosis. Likewise, a study published by Sethi et al. demonstrated that gut microbiome depletion exhibited a significant decrease in tumor burden in subcutaneous models and liver metastatic burden in pancreatic cancer models (33). Beyond simple associations of microbial and disease progression, Pushalkar and colleagues elucidate a direct role for the microbiota in PC progression using preclinical models. Moreover, this study expanded on analyses of the pancreatic tumor microbiome and compared it with the gut microbiome. The authors demonstrated that the cancerous pancreas has a significantly higher burden of bacteria compared with normal pancreas in both mice and humans. When compared to the gut, select bacteria showed differential increase in the tumorous pancreas compared with gut microbiome. To establish a cause-effect relationship, Kras mutant spontaneous PDAC mice (KC, PDX-Cre, LSL-Kras^G12D^) were treated with an oral antibiotic regimen to ablate the pancreatic microbiota. The results indicated that depletion of microbiota delayed tumor progression. Conversely, transferring fecal microbial content from the KPC spontaneous PDAC mouse model (LSL-KrasG12D, LSL-Trp53R172H, and Pdx1Cre), to either germ-free mice or antibiotic-treated KC mice supported pancreatic oncogenesis in both experimental conditions. Looking at the immune microenvironment, bacterial ablation results in a decrease in myeloid-derived suppressor cells, and an increase in M1 macrophages, promoting T-cell activation. They finally showed that PDAC microbiota enable immunosuppression through the activation of TLR (32).

Consistently, using both a xenograft and the KrasG12D/+; PTENlox/+;Pdx1-Cre genetic mouse model of pancreatic cancer, Thomas et al. revealed that antibiotic-treated mice showed lower numbers of malignant lobules than microbiota-intact mice, indicating delayed progression (34). However, unlike the data reported by Pushalkar et al., indicating the existence of intra-tumoral microbes, and its role as a potential instigator of pancreatic carcinogenesis, Thomas et al., were not able to find microbiome composition differences between the cancer and normal samples in their subcutaneous PDAC xenografts. Nevertheless, the same pro-tumor phenotype of gut microbiota was recognized, indicating that the gut microbiota exerts a role in pancreatic carcinogenesis independent of the local tumor microenvironment. Additionally, they noted increased tumor infiltration by TH1 CD4+ T cells and cytotoxic CD8+ T cells after antibiotic-mediated microbiota depletion in NODSCID mice harboring human PDAC xenografts (34). In a similar approach of using antibiotics to ablate bacteria, Sethi and colleagues confirmed that depleting gut microbiota significantly decreased tumor size in a subcutaneous PDAC tumor model. In this study, tumor immunological profiling following bacterial depletion revealed an increase in anti-tumor immune lymphocytic cells (CD3+CD4+IFNγ+, CD3+CD8+IFNγ+, and CD3+IFNγ+), while a decrease in pro-tumorigenic IL17a (IL17a + CD3 +) and IL10(IL10 + CD4 + CD3 +). Notably, the tumor-suppressing effect of gut microbiome depletion was lost when the researchers performed heterotopic implantation in Rag1^–/–^ mice (lacking mature T and B lymphocytes), indicating that the gut microbiota elicits effects by interaction with adaptive immune cells (33).

Employing fecal microbiota transplantation (FMT) from human donors into mice via oral gavage, researchers discovered that intra-tumoral microbial beta-diversity not only exhibited distinct clustering based on the type of FMT administered, but also resulted in a notable reduction in tumor growth in mice that received transplants from long-term survivors compared to those from the other two groups (30). Taken together, these findings suggest that gut microbiome of PDAC models promote disease progression and that bacterial ablation has protective effects. Further, the microbiota of relatively rare long-term PDAC survivors have differential and protective effects against PDAC progression.

## The gut microbiome as a risk factor for pancreatic adenocarcinoma

Certain microbes have been identified as a risk factor for PDAC (35). Among these, the most well-established association is with Porphyromonas gingivalis (P. gingivalis), the intracellular bacterial pathogen responsible for periodontitis. A meta-analysis including eight studies found a relative risk of 1.74 (95% CI, 1.41-2.15) and 1.54 (95% CI, 1.16-2.05) between periodontitis & pancreatic cancer and edentulism & pancreatic cancer, respectively (36). Another analysis using oral mouth wash samples from 361 PDAC patients and 371 matched controls demonstrated a 59% greater risk of PDAC when *P. gingivalis* is present (33, 34). To further elucidate these epidemiological associations, Tan et al. demonstrated accelerated tumor growth in subcutaneous and orthotopic PDAC murine models when exposed to *P. gingivalis* via oral gavage. They confirmed localization of bacteria to the pancreas and a resultant pro-inflammatory TME characterized by neutrophil-dominated milieu with elevated secretion of neutrophil chemokines (CXCL1 and CXCL2) and neutrophil elastase (37). Interestingly, the red complex (*P. gingivalis, treponema denticola, and tannerella forsythia*), major periodontitis-causing pathogens, secrete peptidyl-arginine deiminase (PAD) which is an enzyme known to cause release of neutrophil extracellular traps (NETs). NETs are elevated in both human and murine models of PDAC and associated with poor patient outcomes, metastasis, fibrosis, proliferation and immune evasion (38–40).

In a distinct study, Ye and colleagues explored the potential involvement of *Fusobacterium nucleatum* (F. nucleatum), another oral microbe, in the initiation and progression of PDAC (41). The bacterial load of F. nucleatum was elevated in pancreatic tumors compared to adjacent normal tissues, indicating a potential association with pancreatic oncogenesis. Further experiments demonstrated that F. nucleatum infects pancreatic cells in a Fap2-driven mechanism and stimulates the production of specific cytokines such as GM-CSF, CXCL1, IL-8, and MIP-3α.

These cytokines, namely granulocyte-macrophage colony-stimulating factor (GM-CSF) and C-X-C motif chemokine ligand 1 (CXCL1), are pivotal players in the progression of PDAC. GM-CSF fosters the proliferation of pancreatic cancer cells, fueling their growth and spread within the body. Conversely, CXCL1, a chemokine intimately linked with inflammatory and immune responses, assumes a critical role in facilitating metastasis and conferring resistance to chemotherapy in pancreatic cancer cells (42).

Additional support for this notion is provided by the research conducted by Hayashi and colleagues, revealing that F. nucleatum plays a role in enhancing the aggressiveness of PDAC through alterations in the tumor immune microenvironment. Their implicated mechanism entails F. nucleatum triggering a significant rise in CXCL1 levels. CXCL1, upon binding to the CXCR2 receptor, initiates a series of signaling pathways that notably affect tumor growth and migration. The authors propose that this bacterium exerts its oncogenic role by recruiting MDSCs and suppressing T cells in the tumor microenvironment through the CXCL1-CXCR2 axis in a paracrine manner. This process facilitates immune evasion by the tumor (41, 43). While mechanistic studies on the co-infection of P. gingivalis and F. nucleatum *in vitro* or animal models are lacking, these findings imply that specific bacterial species may drive pancreatic cancer advancement. However, comprehending the impact of individual bacterial species on cancer progression is incomplete without considering the intricate dynamics of microbial interactions. Therefore, associative studies have been conducted to explore the connection between the pancreatic microbiota as a whole and the progression of PDAC, as emphasized by Chakladar et al. (43).

Chakladar et al. revealed a significant link between the intra-pancreatic microbiome composition and key aspects of PDAC progression. They identified 13 microbial species associated with advanced tumor stages, with Acidovorax ebreus notably linked to higher tumor grades. Additionally, nine microbes were correlated with the downregulation of tumor suppressive pathways. The study highlighted associations between specific microbes and immune modulation, with Citrobacter freundii and Pseudomonadales bacterium linked to proinflammatory responses. Moreover, M. hyopneumoniae exhibited dual effects on oncogenic pathways and immune suppression. The presence of A. baumannii and M. hyopneumoniae correlated with smoking-induced genomic alterations, exacerbating PDAC progression (43).

Guo et al. conducted a study examining bacterial communities in various PDAC subtypes, including classic, basal-like, and mixed, using metagenomic sequencing. They identified the basal-like subtype as the most aggressive tumor subtype, characterized by an abundance of specific bacterial species such as Acinetobacter, Pseudomonas, and Sphingopyxis, strongly associated with tumor progression. Further investigation into the functional roles of the basal-like-related microbiome revealed correlations with inflammation pathways and disease progression. Additionally, this microbiome showed positive associations with KRAS signaling, DNA replication, and pathways relevant to pancreatic cancer (44).

## Impact of microbiome on the immune system

**Figure 2:**
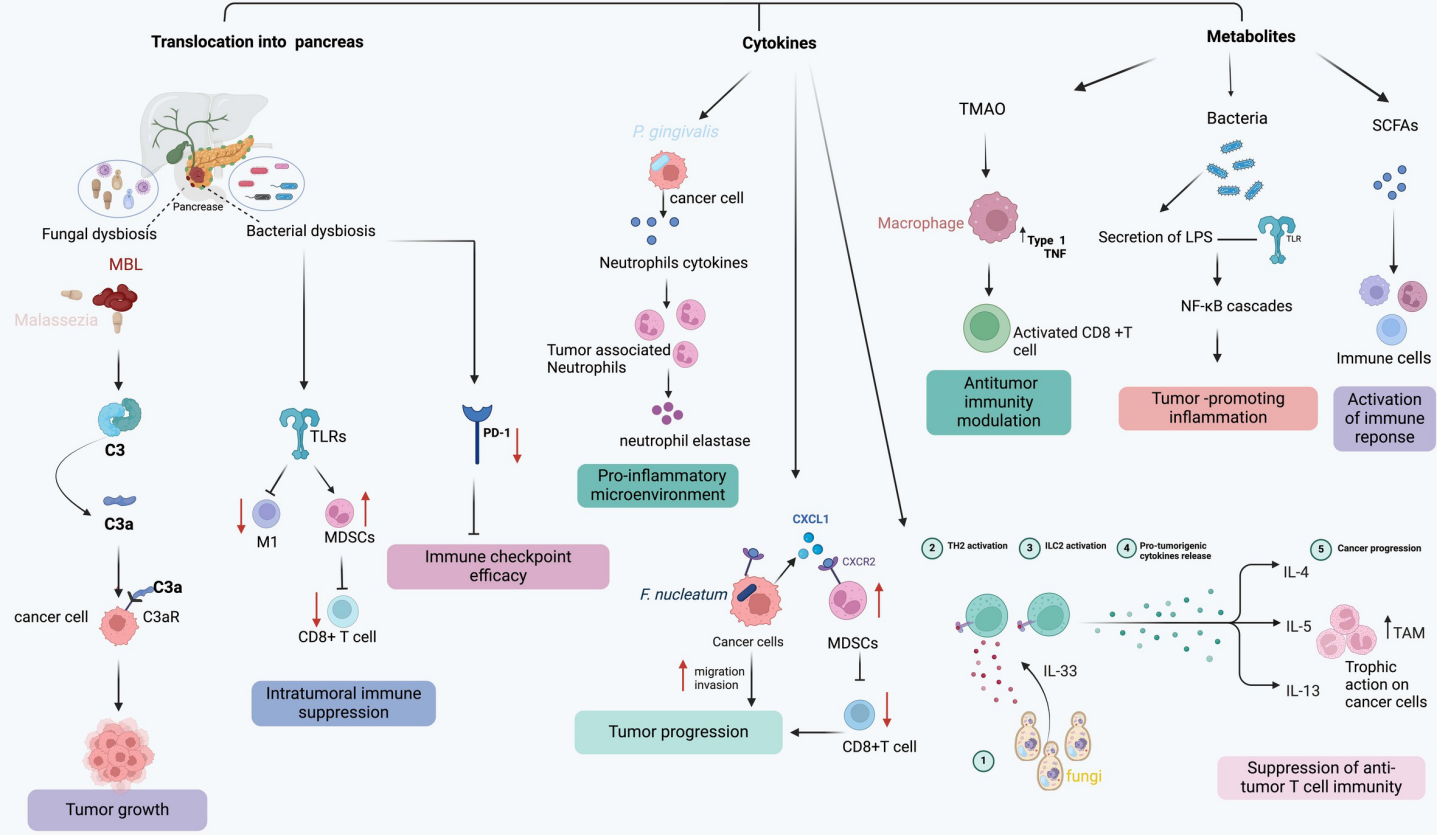
The model demonstrates various mechanisms through which the microbiome influences pancreatic tumor progression and immune response.

The gut microbiome plays a vital role in instructing the host’s adaptive and innate immune systems (45). **Figure 2** highlights the intricate interplay between the microbiome and the immune system at tumorigenesis. Crosstalk between bacteria and the immune system not only occurs along the gut lamina propria but also in extra-intestinal sites (46). PDAC is characterized by an immunosuppressed state both systemically and locally. This immunosuppressive state is correlated with the aggressiveness of the malignancy (47, 48). Insights into the complex alterations that PDAC-related dysbiosis has on immune cell activation and differentiation have been shown in gut microbiome depleted murine models. Treatment of gut microbiome with antibiotics has been showed to increase T-cell infiltration and decrease myeloid-derived suppressor cells (MDSCs), thus implicating PDAC-related dysbiosis in immune evasion. Importantly, lower overall T-cell infiltration has been associated with a poor prognosis while increased intra-tumoral CD8:CD4 T cell ratio has been shown to improve survival as a result of its increased immunogenicity (25, 40, 49, 50).

Long-term survivors of PDAC with the enrichment of aforementioned *Pseudoxanthomonas-streptomyces-Saccharopolyspora* were found to have increased CD8+ T cell infiltration (30). Pancreatic cancer mice with collagen type I knockout, and thus an inability of cancer cells to secrete oncogenic Col1, were found to have a unique intra-tumoral microbiome featuring diminished *Bacteroidales*, increased *Campylobacterales* (51, 52). These mice simultaneously had an increased CD8+ T cell infiltration with reduced tumor progression. Ablation of the microbiome in this model led to a reduced CD8+ T cell infiltration and reduced survival. Conversely, mice gavaged with *P. gingivalis* or *Alternaria alternata* have been shown to have a significant reduction in CD8+ T cell tumor infiltrates (37, 52, 53).

Characterizing the leukocyte subpopulations is also critical as differentiation can have divergent effects on tumorigenesis when the delicate balance of pro- and anti-tumor T cells in the TME is shifted. For example, Th1 CD4+ cells show anti-tumor effects while antigen-restricted Th2 CD4+ T cells promote tumor progression (32, 49, 54). Th2 CD4+ polarization has been shown to be a result of cancer-associated fibroblast mediated secretion of thymic stromal lymphopoietin and correlates to reduced survival (55). Additionally, differentiation of naïve T helper cells into Th17 CD4+ T cell is known to be tumorigenic in multiple cancer types including PDAC (45, 56, 57). Sethi et al. showed that ablation of PDAC microbiome resulted in a significant increase in Th1 CD4+ T cells in TME and a significant decrease in IL-17 and IL-10 secreting T cells. Reduction in tumor growth was not observed when mice were treated with an anti-IL17a antibody (33). Further, accelerated pancreatic intraepithelial neoplasia progression has been seen when IL-17 is overexpressed (56). Similar findings of accentuated Th1 phenotype has been found with microbiome ablation have been observed suggesting the possibility for immunogenic reprogramming and synergistic effects of antimicrobial therapy with immunotherapy as discussed below (32).

## Potential mechanisms of microbiome modulation of immune responses in PDAC

PDAC’s unique tumor immune signature is characterized by a prevalence of immune suppressor cells like tumor-associated macrophages (TAMs), T regulatory (Treg) cells, CD4+ TH2 cells, and myeloid-derived suppressor cells (MDSCs), which collectively hinder the activation, proliferation, and effectiveness of effector T cells, ultimately inhibiting anti-tumor T cell immunity and promoting PDAC progression (58). Studies have reported that microbes in the gut and intrapancreatic can modulate the immunosuppressive intra-tumoral environment in PDAC through innate and adaptive immune response (32). **Table 1** highlights a few potential mechanisms of microbiome modulation of immune responses in PDAC. Several mechanisms have been proposed to explain how microbiota affect immune cells in PDAC microenvironment (59).

**Table 1:** Potential mechanisms of microbiome modulation of immune responses in PDAC.

Mechanism/Model	Description	Microbiota Involved
Microbiome Diversity and Survival	Higher microbiome diversity correlates with longer overall survival in PDAC patients.	Proteobacteria Firmicutes Bacteroidetes
Gut Microbiome Depletion	Manipulating gut microbiome composition in murine models reduces overall tumor burden and fibrosis in PDAC.	Megasphaera Bifidobacterium
Specific Microbial Associations	Porphyromonas gingivalis (P. gingivalis) colonization is linked to an increased risk of PDAC and accelerated tumor growth in murine models.	Porphyromonas gingivalis
Microbiome Influence on Immunity	PDAC-related dysbiosis affects immune cell activation and differentiation, influencing T-cell infiltration and tumor progression.	Campylobacterales P. gingivalis Alternaria
Microbiota Translocation	Gut microbes migrate to the pancreas, activating Toll-like receptors (TLRs) and promoting the expansion of myeloid-derived suppressor cells (MDSCs) and anti-inflammatory M2-like tumor-associated macrophages (TAMs), while inhibiting cytotoxic CD8+ T cells.	Firmicutes Bacteroidetes
Microbial Metabolites	Short-chain fatty acids (SCFAs), such as butyrate, enhance the activity of CD8+ T cells and mitigate the suppression of pro-inflammatory signals in macrophages. Lipopolysaccharides (LPS) activate TLRs, triggering cytokine production and immune evasion.	Firmicutes Bacteroidetes Gram-negative bacteria
Cytokine Secretion	Specific bacterial strains induce the production of interleukin-6 (IL-6), interleukin-10 (IL-10), and transforming growth factor-beta (TGF-β), promoting tumor growth and evading immune surveillance by inhibiting cytotoxic T cells and natural killer (NK) cells.	Firmicutes Bacteroidetes
Fungal Dysbiosis	Fungal abundance increases in cancerous pancreas, with Malassezia spp. implicated in enhancing PDAC progression by binding to mannose-binding lectin (MBL) and activating a complement cascade to evade immune responses.	Malassezia spp.

One proposed model is the translocation of microbiota into the pancreas from the oral or intestinal compartment which initiate important immune alterations (60). This phenomenon was well-illustrated by Pushalkar et al., where they demonstrated how the translocation of specific microbiota contribute to the establishment of an immunosuppressive PDAC tumor microenvironment. Mechanistically, microbiota act by differentially activating selective Toll-like receptors (TLRs) on immunosuppressive monocytic cells, which leads to the expansion of MDSCs and anti-inflammatory M2-like TAMs. These tolerogenic immune populations, in turn, promote the differentiation of suppressive populations of CD4+ T cells while inhibiting the expansion of cytotoxic CD8+ T cells (32).

Another mechanism, which could work in concert with the translocation model, is the release of microbial-derived metabolites, cellular byproducts, or small molecules that influence immune responses, potentially triggering inflammation and contributing to carcinogenesis (60). For instance, short-chain fatty acids (SCFAs) generated by beneficial symbiotic bacteria have been shown to enhance the immune response against tumors, particularly by enhancing the activity of CD8+ T cells (61). Additionally, the SCFA butyrate has the capacity to mitigate the suppression of pro-inflammatory signals in macrophages, such as IL-6, consequently inhibiting tumor growth (62). Unfortunately, in PDAC patients, there is a decline in these beneficial bacteria that produce SCFAs, leading to a diminished antitumor response of CD8+ T cells and indirectly facilitating the tumor-promoting behavior of macrophages (63). In addition, LPS, a component of the outer surface membrane of Gram-negative bacteria, has the capability to bind to TLR4 and TLR2 present on immune cells, subsequently recruiting MyD88 or TRIF adaptor molecules. These molecules, in turn, activate MAPK and NF-κB cascades, triggering the production of inflammatory cytokines that promote cancer cell proliferation (64). Studies have demonstrated that LPS enhances the invasiveness of PDAC cells by activating the TLR/MyD88/NF-kappaB signaling pathway (65). Additionally, these microbial components have the capacity to enter the host systemic circulation to modulate immune response. Yin et al., has demonstrated that disruption of the intestinal barrier triggers high circulating LPS levels and increased LPS accumulation in tumor tissues. In the early stage, LPS infiltration enhances the presence of CD3+ and CD8+ T cells, inhibiting tumor growth. However, prolonged exposure to LPS leads to T cell depletion. Furthermore, LPS induces the upregulation of programmed cell death ligand 1 (PD-L1) through the TLR4/MyD88/AKT/NF-κB pathway and triggers the depletion and apoptosis of tumor-infiltrating lymphocytes (TILs), consequently facilitating cancer immune evasion (66).

In addition to direct interactions and metabolite generation, the tumor microbiome can also influence the immune response by triggering the secretion of several cytokines and chemokines. Particularly, specific bacterial strains can induce the production of interleukin-6 (IL-6), interleukin-10 (IL-10), and transforming growth factor-beta (TGF-β), consequently promoting tumor growth and evading immune surveillance by selectively inhibiting cytotoxic T cells and natural killer (NK) cells (32).

## Fungal dysbiosis in PDAC

Similar to the bacteriome, fungal dysbiosis has been identified within the PDAC TME and is also associated with carcinogenesis via various species-dependent pathways (67). Recent studies have characterized fungal abundance and composition in both human and mouse models, revealing a notable 3000-fold increase in fungi in cancerous pancreas, notably with a high load of Malassezia in PDAC. Aykut et al. reported that targeting fungi with oral amphotericin B treatment provided protection against tumor progression in mouse models of PDAC. The repopulation of antifungal-treated mice with Malassezia globosa, rather than other commensal fungi like Candida, Aspergillus, or Saccharomyces, restored carcinogenesis, confirming Malassezia spp.’s essential role in mediating PDAC progression. Mechanistically, Malassezia species were identified as crucial contributors to oncogenic progression by binding to mannose-binding lectin (MBL) and activating a complement cascade to evade immune responses (67).

Another interesting study comes from Alam and colleagues, who showed that cancer cells produce IL-33, which then triggers the recruitment and activation of TH2 and group 2 innate lymphoid cells (ILC2s), promoting tumor growth in KPC mice. Treatment with amphotericin B led to significant reductions in IL-33 secretion, TH2 and ILC2 cells infiltration, and overall tumor burden. Conversely, administration of either Malassezia globosa or Alternaria alternata by oral gavage into the tumor-bearing mice had the opposite effect, suggesting that fungi could enhance tumor growth by reshaping the TME through IL-33 signaling (53).

## Impact of microbiome in PDAC immunotherapy response

Based on the substantial impact the microbiome can have on PDAC immune response, the microbiota has also been recognized as a potent influencer of the effectiveness of immune checkpoint inhibitors (ICIs) in PDAC (68–70). ICIs operate by inhibiting specific molecules, including programmed cell death protein 1 (PD-1), programmed death-ligand 1 (PD-L1), and cytotoxic T-lymphocyte-associated protein 4 (CTLA-4). These molecules serve as checkpoints that regulate the immune response (71). By blocking these checkpoints, ICIs allow the immune system to recognize and attack cancer cells more effectively, leading to improved anti-tumor immune responses and potentially better outcomes for cancer patients. The manipulation of immune checkpoint inhibitors (ICIs) has become a potent strategy in treating various cancers. CTLA-4 and PD-1/PD-L1 blockade have shown efficacy in melanoma and lung cancer, resulting in improved recurrence-free survival (RFS) and overall survival (OS) (72–74). Additionally, various gastrointestinal cancers, such as hepatocellular, gastric, and esophageal cancers, have also exhibited responsiveness to interventions targeting these pathways (75–77). Despite this, not all cancer patients benefit from ICI immunotherapy. Specifically, fewer than 1% of PDACs respond to ICIs, and a number of phase 1 and 2 clinical trials are currently investigating various combinations of ICIs and traditional chemotherapies (78). The lack of ICB effectiveness in PDAC is thought to be due to low tumor antigen load, limited mutational burdens, inadequate antigen presentation, immune suppression independent of immune checkpoints, and depletion of tumor-specific T cells. Notably, recent studies indicated that non-host factors, such as specific host gut and intratumorally microbes, also play a role in determining efficacy of PD-1 and CTLA4 blocking therapies (75, 76). The interplay between the microbiota and ICI responses has been explored across various cancer models. For example, a study demonstrated that a subcutaneous mouse model of melanoma exhibited enhanced responsiveness to anti-PD-1 treatment upon supplementation with commensal Bifidobacterium (79). These effects were linked to dendritic cell maturation, which result in improved priming and infiltration of CD8+ T cells in the TME. Likewise, Lactobacillus rhamnosus GG demonstrated similar benefits, enhancing responses to anti-PD-1 in melanoma and colon cancer mouse models by triggered type I interferon (IFN) production in dendritic cell, leading to enhancing the cross-priming of antitumor CD8+ T cells (80). In mouse models of PDAC, the eradication of the gut microbiome through antibiotics enhances the efficacy of PD-1-targeted immunotherapy by elevating PD-1 expression (81). In contrast, a recent study by Routy and colleagues found that alterations in gut composition following antibiotic treatment diminish immunotherapy responses in patients with lung, bladder, and kidney cancers (81). These conflicting findings imply that distinct cancer types may induce specific changes in gut and tumor microbiome composition, which can either attenuate or facilitate the function of ICIs (30).

Response to ICIs seems to be closely linked to bacterial metabolites. A recent study conducted by Mirji et al. highlighted the significance of trimethylamine N-oxide (TMAO), a bacterial metabolite, in modulating antitumor immunity, particularly in PDAC. The mechanism involves the activation of type-I interferon gamma pathways, leading to the stimulation of antitumor macrophages and effector T cells within the TME. Moreover, simultaneous administration of TMAO and an ICI demonstrated a synergistic reduction in tumor burden, surpassing the efficacy of either treatment alone. This presents an opportunity not only to enhance antitumor immunity systemically but also to activate it directly from the gut, potentially yielding significant systemic effects (82). LPS has been observed to enhance the efficacy of PD-L1 blockade therapy in murine PDAC models by elevating the expression of PD-L1 (66). Hence, microbiota play an important role in modulating the efficacy of ICIs. However, whether ICIs affect gut microbiota composition requires further investigations.

Understanding the impact of the tumor microbiome on the effectiveness of chimeric antigen receptor T-cell (CAR-T) therapy and other cellular immunotherapies is a burgeoning field of study. CAR-T therapy entails genetically altering a patient’s T cells to express chimeric antigen receptors, empowering them to identify and eliminate cancer cells (83). CAR-T therapy has demonstrated notable efficacy in treating hematological malignancies like B-cell acute lymphoblastic leukemia (B-ALL) and diffuse large B-cell lymphoma (DLBCL). Moreover, it’s being explored as a potential treatment for solid tumors. In addition to CAR-T, other immune cell immunotherapies, such as checkpoint inhibitors, cytokine therapies, and natural killer (NK) cell therapies, are being investigated. These approaches aim to bolster the immune system’s capacity to detect and eradicate cancer cells.

Smith et al. observed that patients with B cell lymphoma and leukemia who harbored higher levels of specific bacterial species such as Ruminococcus, Bacteroides, and Faecalibacterium exhibited a more favorable response to CAR-T cell therapy (84). Furthermore, the study revealed that antibiotic administration, leading to alterations in the gut microbiota, correlated with increased toxicities such as cytokine release syndrome (CRS) and immune effector cell-associated neurotoxicity syndrome (ICANS). These changes were also linked to diminished responses to CAR-T therapy.

Microbiota modulation of CAR-T treatment can be also influenced by microbe-derived metabolites. von Scheidt et al. demonstrated that the bacterial enterotoxin staphylococcal enterotoxin-B (SEB) significantly promoted CAR-T cell proliferation and suppressed solid tumor growth in mice (85). Another recent study highlighted the influence of SCFAs on CAR-T therapy. Luu et al. and colleagues indicated that SCFA enhanced CD25 expression and the secretion of IFN-γ and TNF-α in CAR-T cells targeting tyrosine kinase-like orphan receptor 1 (ROR1), resulting in anti-tumor effects in syngeneic murine melanoma and pancreatic cancer models (61). Both studies underscore the significance of bacterial metabolites in CAR-T therapy. Despite this, the precise mechanisms by which the tumor microbiome impacts the effectiveness of CAR-T cell therapy and other cellular immunotherapies are still not fully understood. More investigation is required to clarify these mechanisms and assess the potential of microbiome modulation to enhance the effectiveness of cellular immunotherapies for pancreatic cancer treatment.

Cancer vaccines and oncolytic virus therapy offer alternative approaches to immunotherapy treatment. Oncolytic vaccines (OVs) are viruses designed to replicate inside tumor cells, destroying them selectively without harming healthy tissue. Additionally, OVs stimulate the immune system’s response to cancer by releasing molecules and antigens, and activating specific T cells. An adapted version, the oncolytic virus vaccine (OVV), specifically targets tumor-specific antigens, thereby increasing its efficacy against tumors (86). These immunotherapy methods present opportunities for tailored and personalized cancer treatment, frequently used alongside other treatments like chemotherapy, radiation, or additional immunotherapies, to enhance patient outcomes (83, 84). Recent research indicates that specific bacteria may substantially impact the efficacy of cancer vaccines and oncolytic virus therapy (87). In the BALB/c-CT26 tumor model, administering Bifidobacterium alongside vaccination resulted in superior protection compared to either vaccination alone or Bifidobacterium administration alone (88). This synergy was also associated with an increase in the frequency of vaccine-specific T cells. Additionally, while OVV-gp33 demonstrated effectiveness in the early stages of CRC, its efficacy decreased in advanced stages due to differences in intestinal microbial composition (86). However, combining probiotics or FMT with OVV-gp33 enhanced its antitumor effect in advanced CRC stages by modulating microbial communities, metabolites, and T-cell activity. While these findings emphasize the significance of the microbiome composition in shaping the effectiveness of cancer vaccines and oncolytic virus therapy, further research is imperative to elucidate the underlying mechanisms comprehensively and explore potential clinical applications.

## Perspectives of microbiome-based therapy in PDAC

**Figure 3:**
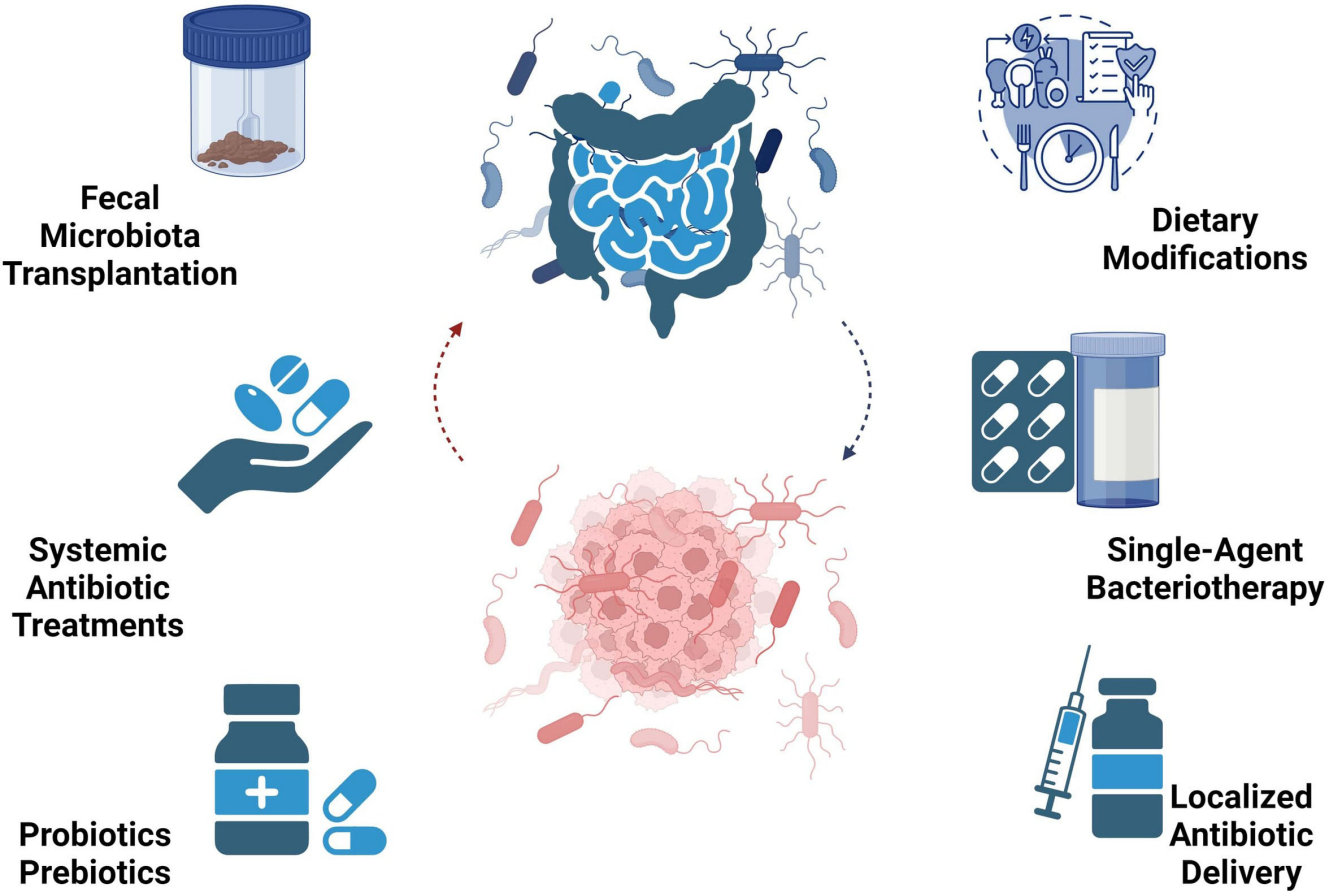
Microbiome-based therapy perspectives in PDAC encompass diverse approaches like FMT, antibiotics, bacteriotherapy, probiotics, prebiotics, and dietary interventions.

The gut microbiota, a promising avenue for PDAC diagnosis and therapy, can be regulated through several methods to therapeutically leverage the emerging research demonstrate a significant role for the microbiome in PDAC pathogenesis. These include fecal microbiota transplantation (FMT), systemic antibiotic treatments for microbiome eradication, localized antibiotic delivery, single-agent bacteriotherapy, probiotics, prebiotics, and dietary modifications. **Figure 3** illustrates microbiome-based therapies in PDAC.

### Fecal microbiota transplantation

Fecal microbiota transplantation (FMT), originating in ancient China during the fourth century, has navigated challenges and sparked extensive technological and theoretical discourse (89). FMT involves transferring stool from a healthy donor into the colon or upper gastrointestinal tract of a recipient with a condition linked to an imbalanced gut microbiome.

FMT can be conducted through various methods, offering flexibility and adaptability to different clinical scenarios (90). These methods include: i) Colonoscopy, where donor fecal material is introduced into the recipient’s colon via a flexible tube inserted through the rectum, enabling widespread distribution throughout the colon; ii) Enema, involving the delivery of fecal material into the rectum using a catheter or syringe, with options for clinical or home-based administration under medical supervision; iii) Nasogastric (NG) Tube insertion, passing through the nose and into the stomach or small intestine, facilitating direct delivery of fecal material into the upper gastrointestinal tract; and iv) Capsules, presenting a non-invasive option with freeze-dried fecal material enclosed, which patients swallow to release the material into the gastrointestinal tract after dissolution in the stomach. These methods offer diverse approaches tailored to patient preferences and clinical requirements.

Initially utilized to address recurrent Clostridium difficile infection (rCDI), the effectiveness of FMT has prompted research into its potential for treating various gastrointestinal and extra-intestinal disorders associated with gut dysbiosis (91).

Recently, there has been a breakthrough in treating refractory ICI-associated colitis with FMT. In a pioneering study, two patients who participated experienced notable alleviation of clinical symptoms during subsequent monitoring (92). In a similar attempt, Tanoue et al. isolated 11 bacterial strains from healthy volunteers’ fecal microbiota, which stimulated the accumulation of interferon-gamma (IFN-γ)-expressing CD8+ T cells in the intestine. These strains demonstrated efficacy in enhancing CD8+ T cell-mediated antitumor immunity, leading to spontaneous immune checkpoint inhibitor treatment and tumor inhibition dependency. Notably, the combination of these 11 strains, predominantly comprising rare and low-abundance species in the normal human microbiota, exhibited significant therapeutic promise against chemotherapy and immunotherapy resistance across a range of cancer types (93, 94). Due to its demonstrated effectiveness in treating metastatic melanoma, FMT holds promise for PDAC patients, offering a potentially safe and effective route to improved prognosis (95).

### Probiotics and prebiotics

Most probiotics on the market are derived from Lactobacillus and Bifidobacterium, along with other lactic acid bacteria, Bacillus sp., Saccharomyces sp., and E. coli. They are available in various forms like fermented foods, supplements, powders, and tablets, including yogurt, cheese, milk, juices, smoothies, kimchi, kombucha, and apple cider vinegar, containing single or mixed probiotic strains. Dietary prebiotics like oligosaccharides (fructans and galactans) are non-digestible food ingredients. They are metabolized by bifidobacteria or host microorganisms into important metabolic products like butyrate, acetate, and propionate, crucial for gut and overall human health (96). Prebiotic oligosaccharides have been investigated for their anti-adhesive properties against pathogens. By mimicking microvillus glycoconjugates, they can interact with bacterial receptors, preventing pathogens from attaching to epithelial cells and inhibiting pathogen colonization. Scientific studies strongly suggest that prebiotics contribute to maintaining a healthy microbiome, thereby enhancing the efficacy and mitigating the side effects of cancer treatments (97, 98). As of now, there are no published reports on the potential links between prebiotics and PDAC. However, Abdul Rahman et al. suggested that prebiotics may exert their effects through a direct mechanism independent of probiotics (99). Oláh et al. utilized a human model to investigate the effects of probiotics, specifically Lactobacillus plantarum 299, in pancreatitis treatment. They found that this bacterium had no adverse effects and contributed to reducing pancreatic sepsis and the need for surgical intervention (100). Recent research suggests that probiotics and their derivatives could offer efficacy against pancreatic cancer while notably decreasing infectious complications post-pancreatoduodenectomy (101).

Ferrichrome, synthesized by Lactobacillus casei ATCC 334, exhibits inhibitory effects on pancreatic, gastric, and colon cancer cells (102). It triggers the activation of M1 TAMs through TLR4-dependent pathways, thereby augmenting the immune response and potentially enhancing the effectiveness of immune checkpoint blocker therapies (103).

In a rat study examining pancreatic tumor cells, A. muciniphila, a Gram-negative bacterium, demonstrated inhibition of tumor cell proliferation by bolstering gut immunity and cytokine release. Additionally, research suggests that probiotics can modulate cancers through various mechanisms, including inducing apoptosis, inhibiting mutagenic activity, downregulating oncogene expression, inducing autophagy, inhibiting kinases, reactivating tumor suppressors, and preventing metastasis (104). These findings imply that probiotics might enhance cancer therapy and immunotherapy effectiveness while minimizing adverse effects.

### Antibiotics

Employing antibiotics in PDAC treatment represents a potentially simple method to alter gut microbiota composition, albeit with variable outcomes. Combining antibiotics with chemotherapy seems to enhance the antitumor effectiveness of chemotherapy and aid in increasing patients’ tolerance to treatment. Weniger et al. observed that certain patients with PDAC did not experience improved progression-free survival (PFS) following adjuvant gemcitabine treatment post-surgery. However, upon identifying Klebsiella pneumoniae in intraoperative bile cultures, patients receiving quinolone treatment exhibited significantly extended survival times (105). However, prolonged antibiotic therapies may lead to side effects, alter the balance between the host and microbiota in all body regions, and result in the selection of antibiotic-resistant bacterial species, thus further studies are essential to evaluate the safety of such strategies. Antibiotic administration either in combination with chemotherapeutics or immunotherapeutic strategies appears to shorten overall survival (106). Antibiotics are often ordered to cancer patients before or during immunotherapy, which can cause significant changes in the gut microbiome leading to dysbiosis (107).

In a retrospective analysis involving 148 metastatic PDAC patients, the utilization of macrolide antibiotics for over 3 days during treatment correlated with extended progression-free survival (PFS) and overall survival (OS) (108). On the contrary, certain studies have suggested a possible association between antibiotics and reduced OS. Hasanov et al. found that tetracycline usage was significantly linked to shorter survival in resected PDAC patients compared to other antibiotics (such as quinolones, beta-lactams, nitroimidazoles, glycopeptides, macrolides) administered. Additionally, there was a trend towards shorter PFS in patients with resectable PDAC (109). Other studies have unveiled the role of gut microbiota in immunogenic reprogramming within the tumor microenvironment, inhibiting tumor growth through anti-tumorigenic T-cell activation. Furthermore, antibiotics have been shown to enhance immune response and increase sensitivity to immunotherapy, suggesting their potential utility in combination therapeutic strategies (110).

### Dietary modification

**Figure 4:**
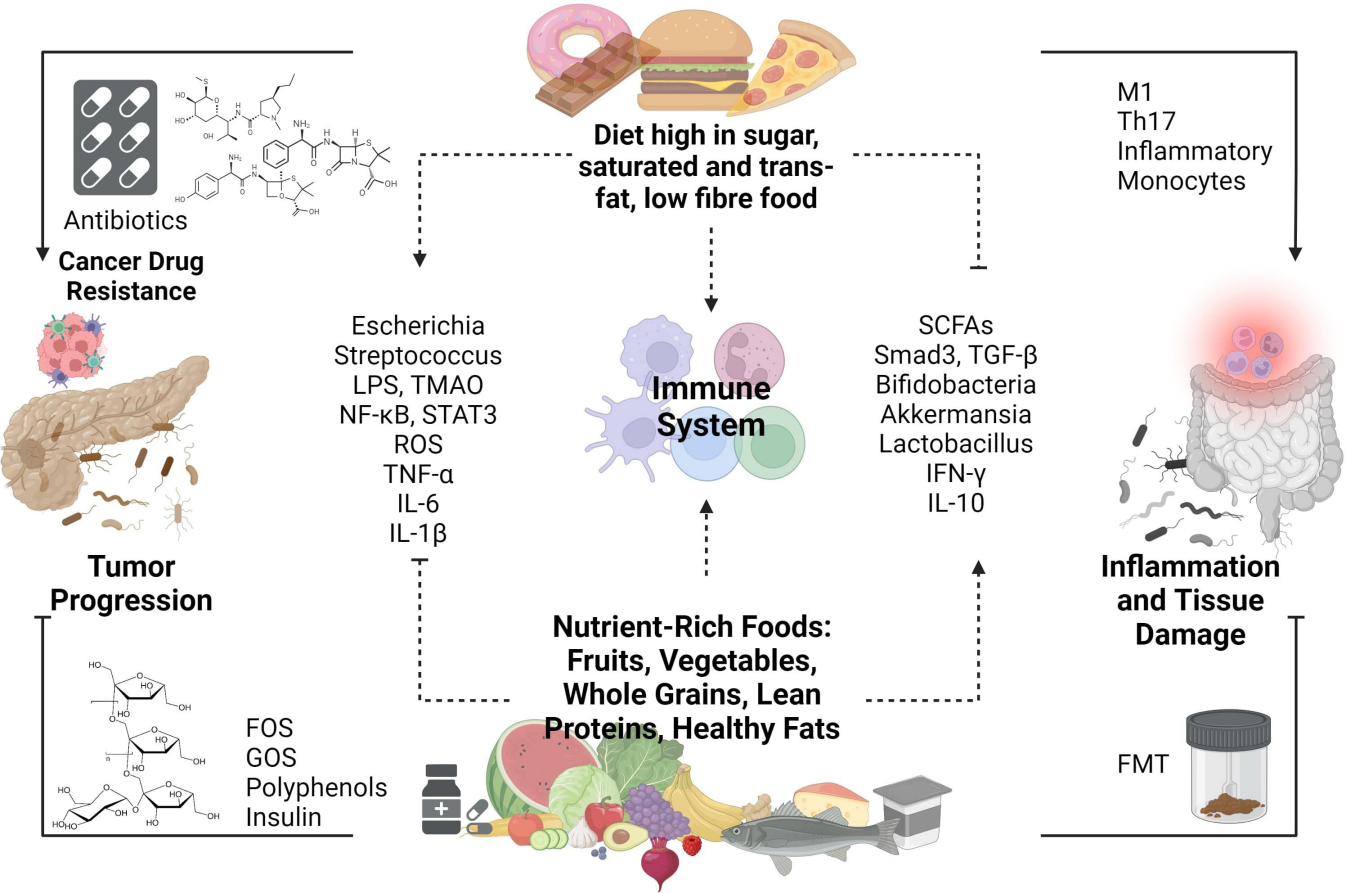
Impact of Dietary Composition on the Dynamic Interplay Between the Gut Microbiome, Immune Responses, and Pancreatic Ductal Adenocarcinoma (PDAC).

Dietary intake significantly shapes the gut microbiota composition (see **Figure 4**). Macronutrients (carbohydrates, fats, proteins), micronutrients (minerals, vitamins, trace elements), and polyphenols all play pivotal roles in influencing the quality and quantity of gut microbiota (111). It is known that enhancing NK-cell antitumor immunity and restraining tumor growth in PDAC models can be achieved by blocking VB6-dependent one-carbon metabolism (111). A high-fiber diet significantly impacts the composition and quantity of gut microbiota in the intestines. Dietary fiber, which can only be broken down and fermented by gut microbiota enzymes, results in the production of acetate, propionate, and butyrate. This fermentation process lowers the colon’s pH, influencing the survival of microbiota species in the acidic environment and inhibiting harmful bacteria such as Clostridium difficile (112).

SCFA concentrations are predominantly influenced by diet and gut microbiota. Research suggests that SCFAs play diverse roles in health, including enhancing immune cell activity and regulating glucose and cholesterol levels. Poor fiber diets, associated with decreased SCFA concentrations, have been linked to increased cancer and inflammatory disease risks, particularly in breast and gastric cancers (113). SCFAs exert anticancer effects by inhibiting cell growth and migration, suppressing histone deacetylase activity, and inducing apoptosis. These actions contribute to the prevention and treatment of gastrointestinal and lung cancers (114). The regulation of gut microbiota has a direct or indirect impact on SCFA concentration, offering potential strategies for cancer treatment. The interaction between SCFAs and TGF-β illustrates the beneficial effects of dietary fiber in colon cancers. TGF-β activates SMAD3 upon binding to its receptors on gut epithelial cells. Butyrate, a type of SCFA, also influences gut epithelial cells and enhances SMAD3 expression. SMAD2 and SMAD3, members of the receptor-regulated SMADs (R-SMAD) family, serve as substrates for TGF-β receptors. Upon phosphorylation, SMAD2 and SMAD3 form complexes with SMAD4, facilitating nuclear translocation. Within the nucleus, the SMAD complex regulates the expression of targeted genes (115). Foods that support increased levels of SCFA are indigestible carbohydrates and fibers such as inulin, resistant starches, gums, pectins, and fructo-oligosaccharides.

Undigested food components undergo metabolism to produce a diverse array of metabolites. Dietary fiber, a key source of undigested food components, consists of organic compounds resistant to digestion and absorption in the human small intestine, and its increased intake as part of a healthy diet has shown beneficial effects on PDAC (116). Conversely, excessive alcohol consumption, a risk factor for development of PDAC, can influence gut microbiota, particularly enriching families like Verrucomicrobia, Actinobacteria, and Proteobacteria, while reducing Firmicutes and Lactobacillus. This imbalance, along with the production of acetaldehyde, a significant metabolite of ethanol, can induce inflammation, potentially leading to chronic pancreatitis and subsequent cancer development (117).

Various cancers exhibit altered cholesterol metabolism, and recent studies suggest that manipulating systemic cholesterol levels could enhance immunotherapy responses. However, cholesterol’s influence on inflammation is complex in mammals, with both proinflammatory and anti-inflammatory effects observed across different immune cell types and contexts (118). By employing metabolic subtyping techniques, researchers have identified distinct metabolic profiles that offer insights into potential therapeutic strategies (119). For instance, one subtype characterized by heightened glycolytic activity tends to be associated with poorer survival rates among pancreatic cancer patients. In contrast, another subtype marked by increased cholesterogenic activity appears to be linked with more favorable outcomes, potentially attributable to higher energy expenditure.

Both the quantity and type of fats consumed significantly influence the gut microbiota. Diets high in saturated and/or total fats consistently exhibit adverse effects on the intestinal microbiome. For instance, saturated fats diminish beneficial microbes like Bifidobacterium and Fecalibacterium, elevating the Firmicutes to Bacteroides (F/B) ratio. Conversely, unsaturated fats reduce detrimental microbes such as Escherichia and Streptococcus species while boosting beneficial bacteria like Bifidobacteria and Akkermansia, thereby lowering the F/B ratio (120). It is known that a high-fat diet depletes the abundance of beneficial microbes, but transitioning to a low-fat diet can reverse this effect. Indeed, a high-fat, high-energy diet facilitates the absorption of harmful microbial metabolites, such as bacterial LPS, into the bloodstream (121). This phenomenon may occur due to microbiota’s influence on carbohydrate metabolism and SCFA production, which can compromise the integrity of intestinal mucosal epithelium tight junctions, allowing bacterial endotoxins to enter circulation.

## Future frontiers in microbiome research

Microbiome research has undergone remarkable advancements, unveiling the intricate interplay between microbial communities and human health. Venturing into the future, new horizons beckon, offering exciting prospects for further exploration and discovery. A key area for future investigation lies in unraveling the dynamic nature of microbial communities and their response to environmental stimuli. Understanding how microbiomes evolve over time, particularly in the context of factors like diet, lifestyle, and environmental exposures, can provide invaluable insights into their role in health and disease.

Another promising avenue is the elucidation of host-microbiome interactions and their influence on human physiology. By delving into the molecular mechanisms underlying these interactions, researchers can uncover how microbes shape host immune responses, metabolism, and overall well-being, paving the way for innovative therapeutic interventions. Beyond taxonomy, future research efforts will likely focus on functional characterization of microbial communities. By deciphering the metabolic pathways and gene expression profiles of microbiomes, scientists can uncover key metabolic signatures and potential therapeutic targets, shedding light on the intricate workings of these complex ecosystems. Furthermore, advancements in technology will continue to drive progress in microbiome research. Cutting-edge techniques such as high-throughput sequencing, single-cell analysis, and computational modeling offer unprecedented opportunities to study microbial communities with unparalleled resolution and depth, propelling the field forward into new realms of discovery.

Translating microbiome research into clinical applications represents a burgeoning frontier. From personalized probiotics tailored to individual microbiome profiles to microbiome-based therapeutics targeting a range of diseases, including metabolic disorders, autoimmune conditions, and cancer, the potential for harnessing the power of microbial communities for therapeutic benefit is immense. Moreover, exploring the influence of steatorrhea on the gut microbiome presents an intriguing avenue for future investigation specific to pancreatic cancer. Steatorrhea, characterized by the malabsorption of fats and subsequent lipid excretion, may profoundly impact microbial composition and function in the gut. Alterations in the relative abundance of bacterial taxa, including Firmicutes, Bacteroidetes, and Actinobacteria, have been reported in individuals with steatorrhea (122). Understanding these interactions could unveil novel insights into how steatorrhea after pancreatectomy could influence oncologic outcome and inform targeted therapeutic strategies.

## Conclusions

The gut microbiome’s role in health, including cancer, is significant, with dysbiosis implicated in disease. Altered microbiota composition affects oncogenesis, immune response, and treatment outcomes. Greater microbiome diversity correlates with prolonged survival in PDAC patients, suggesting prognostic biomarkers. Murine models demonstrate the gut microbiota’s direct impact on PDAC progression, with fecal microbiota transplantation showing promise in mitigating it.

Specific microbial species, like Porphyromonas gingivalis and Fusobacterium nucleatum, promote oncogenesis by modulating the tumor microenvironment and immune responses. They contribute to tumor growth, metastasis, and therapy resistance. The broader pancreatic microbiome is associated with key aspects of PDAC progression, underscoring the complex interplay in disease pathogenesis.

The immune system’s role in PDAC is pivotal, influenced by dysbiosis within the tumor microenvironment. Gut and tumor microbiome manipulation alters immune cell infiltration and function, affecting tumor progression and therapeutic response. Fungal dysbiosis further influences PDAC progression, highlighting the microbiome’s multifaceted role in shaping disease outcomes.

The microbiome significantly influences immunotherapy approaches in PDAC, impacting treatment outcomes. Various methods, including fecal microbiota transplantation, probiotics, prebiotics, dietary modifications, and antibiotics, can regulate the gut microbiota. These approaches reshape the tumor microenvironment and enhance treatment outcomes, with dietary modifications offering potential anticancer effects. Personalized PDAC therapy opportunities arise from understanding and manipulating the microbiome, necessitating further research to optimize interventions and explore combination therapies.

## Author contributions

ET: Conceptualization, Data curation, Formal analysis, Funding acquisition, Investigation, Methodology, Project administration, Resources, Software, Supervision, Validation, Visualization, Writing – original draft, Writing – review & editing. FP: Writing – original draft. GM: Writing – original draft. MS: Writing – original draft. SJ: Writing – original draft, Writing – review & editing. BB: Conceptualization, Data curation, Formal analysis, Funding acquisition, Investigation, Methodology, Project administration, Resources, Software, Supervision, Validation, Visualization, Writing – original draft, Writing – review & editing.
